# A zebrafish model of growth hormone insensitivity syndrome with immune dysregulation 1 (GHISID1)

**DOI:** 10.1007/s00018-023-04759-y

**Published:** 2023-03-30

**Authors:** Somayyeh Heidary, Nagendra Awasthi, Nicole Page, Theo Allnutt, Rowena S. Lewis, Clifford Liongue, Alister C. Ward

**Affiliations:** 1 School of Medicine, Deakin University, Pigdons Road, Geelong, VIC 3216 Australia; 2 School of Life and Environmental Sciences, Deakin University, Burwood, VIC 3125 Australia; 3 IMPACT, Deakin University, Geelong, VIC 3216 Australia

**Keywords:** STAT5, GHISID, Growth, Growth hormone, Immunity, Zebrafish

## Abstract

Signal transducer and activator of transcription (STAT) proteins act downstream of cytokine receptors to facilitate changes in gene expression that impact a range of developmental and homeostatic processes. Patients harbouring loss-of-function (LOF) *STAT5B* mutations exhibit postnatal growth failure due to lack of responsiveness to growth hormone as well as immune perturbation, a disorder called growth hormone insensitivity syndrome with immune dysregulation 1 (GHISID1). This study aimed to generate a zebrafish model of this disease by targeting the *stat5.1* gene using CRISPR/Cas9 and characterising the effects on growth and immunity. The zebrafish Stat5.1 mutants were smaller, but exhibited increased adiposity, with concomitant dysregulation of growth and lipid metabolism genes. The mutants also displayed impaired lymphopoiesis with reduced T cells throughout the lifespan, along with broader disruption of the lymphoid compartment in adulthood, including evidence of T cell activation. Collectively, these findings confirm that zebrafish Stat5.1 mutants mimic the clinical impacts of human STAT5B LOF mutations, establishing them as a model of GHISID1.

**Supplementary Information:**

The online version contains supplementary material available at 10.1007/s00018-023-04759-y.

## Introduction

The signal transducer and activator of transcription (STAT) proteins are a family of transcription factors that regulate gene transcription in response to cytokines and other extracellular signals [1]. Two closely-related STAT proteins, STAT5A and STAT5B, are stimulated by a wide variety of cytokines including growth hormone (GH), prolactin (PRL) and a range of interleukins (ILs) [2]. GH utilises STAT5B to facilitate much of its effects on somatic growth [3] and sexual dimorphism [4], PRL employs STAT5A to stimulate mammopoiesis and lactogenesis [5], with IL-2 and related cytokines engaging both STAT5B and STAT5A to regulate lymphocyte development and function [6].

Growth hormone insensitivity syndrome (GHIS) represents a group of rare genetic disorders characterised by postnatal growth failure due to functional insulin-like growth factor (IGF)-1 deficiency despite normal or elevated serum GH [7]. GHIS may be acquired through chronic illness or due to genetic defects in components of the GH–IGF-1 axis [8]. The latter include mutations impacting the GH receptor (GHR) [9], IGF-1 [10], the IGF binding protein IGFALS [11], the regulator of IGF-binding proteins PAPPA2 [12] as well as STAT5B [13]. The STAT5B mutations additionally result in immune defects, particularly impacting T lymphocyte development, with this multi-faceted disorder referred to as GHIS with immune dysregulation (GHISID) [7]. Two types have been identified, an autosomal-recessive form due to loss-of-function (LOF) STAT5B mutations, termed GHISID1 [14–16], and a less severe autosomal-dominant form due to alternate STAT5B lesions, termed GHISID2 [17].

Zebrafish represents a powerful animal model to mimic human inherited diseases that has been widely exploited to study growth and immunity [18, 19]. Moreover, there is high conservation of cytokine signalling pathway components between zebrafish and mammals, including cytokine receptors [20] and STAT proteins [21]. Two *STAT5*-related genes have been identified in zebrafish, with *stat5.1* most similar to *STAT5B* [22]. This study used genome editing to target *stat5.1* and generate a zebrafish model of GHISID1 that was characterised throughout the life-course.

## Materials and methods

### Fish husbandry and genetic manipulations

Zebrafish were maintained using standard husbandry practices [23], following national guidelines for their care and use, with all studies approved by the Deakin University Animal Ethics Committee. Synthetic guide RNA (gRNA) targeting exon 5 and 16 of the *stat5.1* gene were generated using Zifit to design relevant oligonucleotides (5'-TAGGAGCTGCGAATACTGACTC and 5'-AAACGAGTCAGTATTCGCAGCT, 5'-TAGGAGCGGATAGAGAAGTCTT and 5'-AAACAAGACTTCTCTATCCGCT, respectively) as described [24]. Wild-type (WT) embryos were injected with 12.5 ng/μl gRNA and 100 ng/μl Cas9 mRNA (Sigma), raised to adulthood and out-crossed with WT fish. Identified carriers of *stat5.1* mutant alleles were again out-crossed with WT fish to produce heterozygotes that were in-crossed to generate a homozygote mutant line.

### Genomic DNA analysis

Genomic DNA from adult fin clips and whole embryos was isolated with QuickExtract following the manufacturer’s instructions. This was subjected to polymerase chain reaction (PCR) with *stat5.1* specific primers for exon 5 (5'-GTGGGCGGGTTAATGGACAG, 5'-TACACGCATACCCTGTATTCTGAG) and 16 (5'-GTAACCATTTTAAAGCATCT, 5'-GTAACCATTTTAAAGCATC) for high resolution melt (HRM) analysis [25] to identify potential mutants, which was confirmed with gel electrophoresis using the same primers for exon 5 and an alternate downstream primer for exon 16 (5'-GTGTAGTATTTGGAGAA) and ultimately by Sanger sequencing at the Australian Genome Research Facility.

### Body measurements

Embryos and juvenile fish were imaged with a MVX10 monozoom microscope using a 1 × MVXPlan Apochromat lens (NA = 0.25) and DP74 camera using CellSens Dimension 1.6 software (Olympus) and length determined using ImageJ software, with growth rates derived from that data. Adult fish was weighed and imaged using a camera phone (Apple iPhone 6, 8-megapixel, 1/3-inch sensor, 1.5 µm pixel size) next to a ruler, with standard length measured from snout to caudal peduncle [26].

### Lipid analysis

Euthanized larvae were stained with 10 μg/ml Nile Red (Sigma) solution for 30 min at 28°C in the dark [27], and imaged with a MVX10 fluorescence microscope at an excitation wavelength of 488 nm using CellSens Dimension 1.6 software (Olympus) with the area of staining quantified using ImageJ software. Total lipid was extracted and quantified from adult fish as described [28].

### qRT^2^-PCR

Total RNA was extracted from juvenile zebrafish with TRIsure™ (Bioline) and dissected adult tissues with an RNeasy Mini Kit (Qiagen) according to the manufacturers’ protocols. RNA samples were subjected to quantitative real-time reverse transcription PCR (qRT^2^-PCR) with primers for the zebrafish genes *actb* (5'-TGGCATCACACCTTCTAC, 5'-AGACCATCACCAGAGTCC), *cd79a* (5'-GCGAGGGTGTGAAAAACAGT, 5'-CCCTTTCTGTCTTCCTGTCCA), *cd8* (5'-ACTCTTCTTCGGAGAGGTGAC, 5'-ACAGGCTTCAGTGTTGTTTGAA), *ctla4* (5'-GGGAACGGCACTGTTGTTTAC, 5'-TGTCTGGCTCTTGCTTTGAC), *fasn* (5'-CATATTCTGGGTGTGCGTGAC, 5'-GCTTTACAGGAGACTCCTCTTTC), *gh1* (5'-TCTTATGCCTGAGGAACGC, 5'-AGGTCTGGCTGGGAAACTC), *igf1* (5'-CCGCATCTCATCCTCTTTCTC, 5'-CCTGTCTCCACACACAAACTGC), *igf2a* (5'-AGTGTCACAGGCTCTTCACAAG, 5'-GATGGGACTCCTCTCCTTAACC), *igf2b* (5'-TTCTGTTTGCCATACCTGCTC, 5'-ATCCCACGATTTTGAGAACG), *igf3* (5'-GCGGACGAGAACTAGTGGAC, 5'-ATGCCTTTCCCACGAGAGC), *ighm* (5'-CCGAATACAGTGCCACAAGC, 5'-TCTCCCTGCTATCTTTCCGC), *ikzf4* (5'-ATTGCAATGGCCGTTCGTAC, 5'-ATGGAGTTAGCACTGAGTGAGC), *nklb* (5'-TGGGAGGTTCGTGAAGTGG, 5'-CACTTTAGTACAGATGGTGTTTGGTC), *nkld* (5'-TGGTGAAATCCCAACAGAGCA, 5'-TTTCATCCTGAGTTGCACCA), *prl* (5'-TGAAGTGCCGGAGGATGA, 5'-ACGGGAGAGTGGACAGGTTGT), *srebf1* (5'-GAAGCTAAGCTCAATAAGTCTGC, 5'-TCAGAGACTTGTTCTTCTGGATG), *stat5.1* (5’-CATTACGCTTCACAGCTAAAGAGC, 5’-GTTTATCTGGTGGTATTTCTGTGAC) and *tcra* (5'-ACTGAAGTGAAGCCGAAT, 5'-CGTTAGCTCATCCACGCT) and Sensifast SYBR Lo-Rox qRT^2^-PCR kit (Meridian Biosciences) using an AriaMx Real-time PCR System. Data were normalised to *actb* and fold-change was calculated using the ΔΔCt method [29].

### Whole-mount in situ hybridisation (WISH)

Anaesthetised embryos were fixed in 4% (w/v) paraformaldehyde at 4°C before WISH with anti-sense digoxigenin-labelled gene-specific probes, as described [30]. These were imaged on a MVX10 monozoom microscope using a 1 × MVXPlan Apochromat lens (NA = 0.25) with a DP74 camera with the area of staining captured using CellSens Dimension 1.6 software (Olympus) and quantified using ImageJ software.

### Histological analysis

Adult peripheral blood smears and kidney marrow cytospin preparations were stained with Giemsa (Sigma). These were viewed on a Leica DM E microscope with a 100 × oil objective (NA = 1.25) to perform manual white blood cell differential counts and imaged using a SC50 camera using CellSens Dimension 1.6 software (Olympus).

### Statistical analysis

Statistical analysis was performed with GraphPad Prism (Version 8) software using the unpaired independent student’s *t*-test, with Welch’s correction where appropriate or two-way ANOVA when considering both genotype and sex. The level of statistical significance was denoted as follows: ns: not significant; significant difference between genotypes: *: *p* < 0.05, **: *p* < 0.01, ***: *p* < 0.001; significant difference between sexes: ^#^: *p* < 0.05, ^##^: *p* < 0.01, ^###^: *p* < 0.001.

### RNAseq analysis

Total RNA was extracted from 5 dpf embryos using an RNeasy Plus Mini kit (Qiagen) following the manufacturer’s instructions. RNA quality was confirmed using an Agilent 2100 bioanalyzer, and an NGS library prepared using a TruSeq RNA library Prep Kit v2 (Illumina). Sequencing RNAseq was performed using a HiSeq 2500 system (Illumina) with 100 bp single end reads, which were trimmed to remove Illumina adaptor sequences and low quality ends using Trimmomatic v0.36 [31]. Trimmed reads were mapped to the coding sequences (cds) of the *Danio rerio* reference genome (Build 11) using BBmap v38.3. Mapped read counts per sample were tabulated using a custom Python script and statistically analysed for differential expression using EdgeR [32] and ALDEX2 [33]. The PANTHER classification system version 17 (http://www.pantherdb.org/) was used to determine the pathways in which gene expression was altered [34].

## Results

### Generation of zebrafish LOF Stat5.1 mutant

**Fig. 1:**
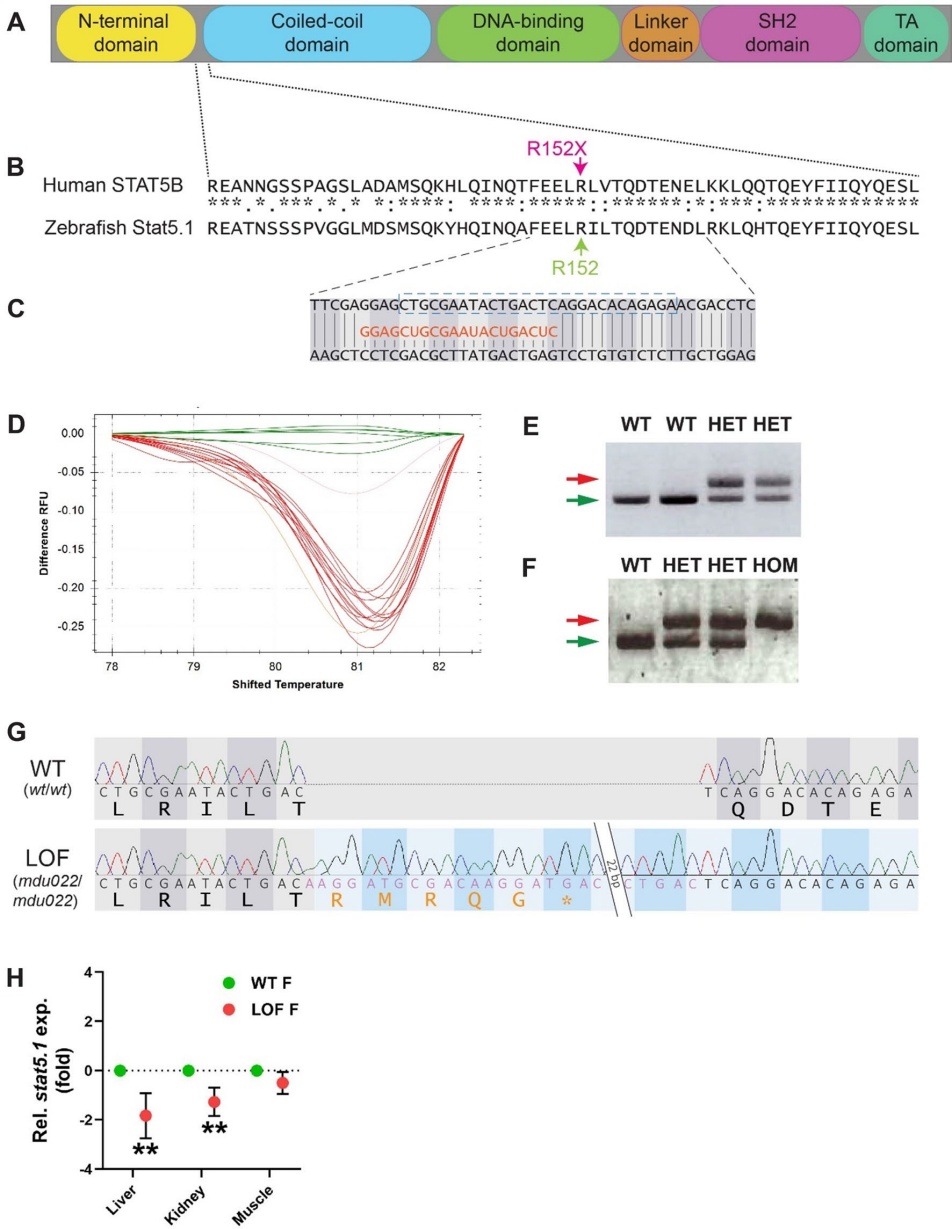
Generation of a zebrafish Stat5.1 mutation mimicking those in human STAT5B associated with GHISID1. **A** Schematic diagram of the STAT5B protein domain architecture consisting of N-terminal (yellow), coiled-coil (blue), DNA-binding (green), linker (orange), SH2 (pink), and transactivation (TA) (dark green) domains. **B** Alignment of human STAT5B with zebrafish stat5.1 sequences around the site of the R152X mutation (green) seen in human GHISID1, showing identical (*), highly similar (:) and similar (.) amino acids. **C** Nucleotide sequence of zebrafish *stat5.1* genomic DNA targeted using CRISPR/Cas9 with the gRNA (orange) shown and the coding phase indicated. **D** HRM analysis of a sample of embryos injected with *stat5.1* gRNA and Cas9, with wild-type embryos in green and other colours representing potential mutants. **E–F** Genotyping by PCR and gel electrophoresis of representative F1 wild-type (WT) and heterozygous (HET) mutant carriers (**E**) and F3 WT, HET and homozygous (HOM) fish (**F**), with the wild-type allele indicated with the green arrow and the mutant allele with red. **G** Sequence of homozygous wild-type (WT, *wt/wt*) and loss-of-function (LOF, *mdu022/mdu022*) mutant *stat5.1* showing the chromatogram along with corresponding nucleotides, including a 47 bp insertion (pink text) in the LOF mutant. The coding phases and protein translations are shown, including the alternate phase and resultant five de novo amino acid residues and stop codon (orange text) following T155 in the LOF mutant. **H** Expression analysis of *stat5.1* using qRT.^2^-PCR on the indicated tissues from WT and LOF adult female (F) zebrafish. Data was normalised to *actb* and represented as relative fold-change compared to WT, with mean ± SD shown and statistical significance indicated (*n* = 6)

Like other STAT proteins, STAT5B consists of multiple domains, specifically the N-terminal, coiled-coil, DNA binding, linker, SH2 and transactivation domains [1] (Fig. 1A). A variety of autosomal-recessive loss-of-function (LOF) STAT5B mutations have been described [13], including R152X that truncates after the N-terminal domain in a region well-conserved between human STAT5B and zebrafish Stat5.1 (Fig. 1B), which was chosen for targeting by CRISPR/Cas9-mediated genome editing [35].

Embryos were injected with gRNA targeting the corresponding region of the *stat5.1* gene (Fig. 1C) as well as Cas9 mRNA. High resolution melt (HRM) analysis identified multiple mutagenic events in a sample of injected embryos (Fig. 1D) and so the remainder were raised to adulthood and out-crossed with wild-type (WT) fish and progeny carrying a potential *stat5.1* mutation identified by genotyping (Fig. 1E). These carriers were again out-crossed with WT fish, and heterozygous progeny in-crossed with homozygous mutants identified (Fig. 1F) that were characterised by sequence analysis. This identified the mutant allele *mdu022*, which represents a 47 bp insertion that results in the addition of five de novo amino acid residues after T155 followed by a stop codon (Fig. 1G), resulting in a large truncation of Stat5.1 similar to that observed with the human STAT5B R152X LOF mutation [16]. Expression analysis confirmed a large reduction in *stat5.1* expression in key tissues, including the liver and kidney, the latter being the major site of adult zebrafish haematopoiesis [36], but not muscle (Fig. 1H).

### LOF Stat5.1 mutation impacts growth and adiposity

**Fig. 2:**
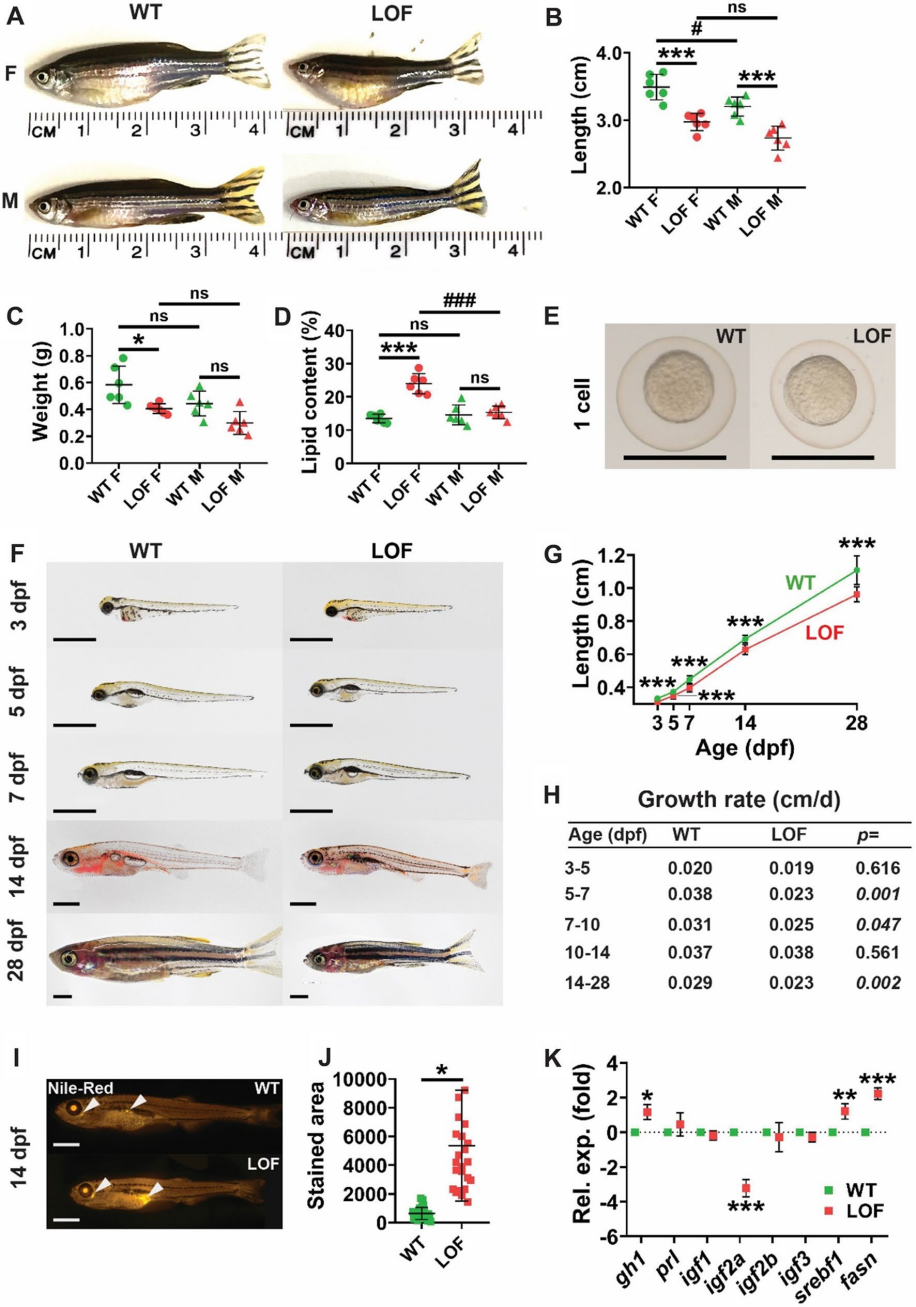
Effect of LOF Stat5.1 mutation on growth and adiposity. **A–D** Assessment of adult size and body composition in female (F) and male (M) homozygous *stat5.1*^*wt/wt*^ wild-type (WT) and *stat5.1*^*mdu022/mdu022*^ loss-of-function (LOF) mutants at 5 months post-fertilisation (mpf), presenting representative images **(A)** along with quantification of standard length **(B)**, wet weight **(C)** and lipid content **(D),** showing values for individual fish as well as mean ± standard deviation (SD) with statistical significance indicated (*n* = 6). **E–H** Growth trajectory during early life, presenting light microscopic images of representative WT and LOF individuals at the 1 cell stage **(E)** and at the indicated times post-fertilisation **(F)**, along with quantification of length showing mean ± SD and statistical significance (*n* = 10–33) **(G)** and relative growth rate in the indicated age ranges calculated from these data with *p*-values shown **(H)**. Scale bar = 1 mm. **I–J** Lipid analysis in juveniles, presenting fluorescent images of representative 14 dpf WT and LOF stained with Nile Red **(I)** and quantification of the area of Nile Red staining showing individual values as well as mean ± SD, with statistical significance indicated (*n* = 24) **(J)**. Scale bar = 1 mm. **K** Expression analysis of growth and metabolic genes in 28 dpf juveniles **(K)** using qRT^2^-PCR on WT and LOF samples. Data for the indicated genes were normalised to *actb* and represented as relative fold-change compared to WT, with mean ± SD shown and statistical significance indicated (*n* = 6)

Both female and male adult *stat5.1*^*mdu022/mdu022*^ LOF mutant zebrafish were clearly smaller than their respective WT counterparts (Fig. 2A), with significantly reduced standard body length in both sexes (Fig. 2B) and wet weight in females (Fig. 2C). In contrast, female but not male LOF fish displayed increased adiposity compared to respective WT fish as judged visually (Fig. 2A) and by analysis of lipid content (Fig. 2D). Female LOF fish also showed significantly increased lipid content compared to male LOF fish, a difference not evident between female and male WT fish (Fig. 2D), whereas the increased length of female compared to male WT fish was not evident in LOF fish (Fig. 2B).

To further investigate these differences, individuals were closely monitored throughout the life-course. Eggs from LOF parents were found to be smaller in size than those from WT parents (Fig. 2E), which reached statistical significance (WT: 0.67 ± 0.029 mm, LOF: 0.64 ± 0.027 mm, *p* = 0.028). The reduction in size continued from 3 to 28 days post-fertilisation (dpf) (Fig. 2F–G), with close analysis revealing a significantly decreased growth rate in LOF individuals between 5–7, 7–10 and 14–28 dpf (Fig. 2H). Relative adiposity was also assessed by visualisation of lipid droplets using Nile Red, which identified a significant increase in lipid accumulation in 14 dpf LOF larvae (Fig. 2I–J).

To consider potential molecular pathways involved in the observed growth deficiency and increased adiposity, 28 dpf juveniles were analysed for the expression of genes specific for growth and lipid metabolism (Fig. 2K). A significant increase in expression of growth hormone (*gh1*) [37] was observed, while that of prolactin (*prl*) [38] was unaltered in LOF compared to WT fish. Amongst the four zebrafish *igf* genes [39], there was a significantly decreased expression of *igf2a* but not the other *igf* genes. Moreover, expression of the lipid-metabolism related genes, *fasn*, encoding fatty acid synthase [40], and *srebf1*, involved in hepatic lipogenesis [41], was significantly increased in LOF fish.

### LOF Stat5.1 mutation impacts lymphopoiesis

**Fig. 3:**
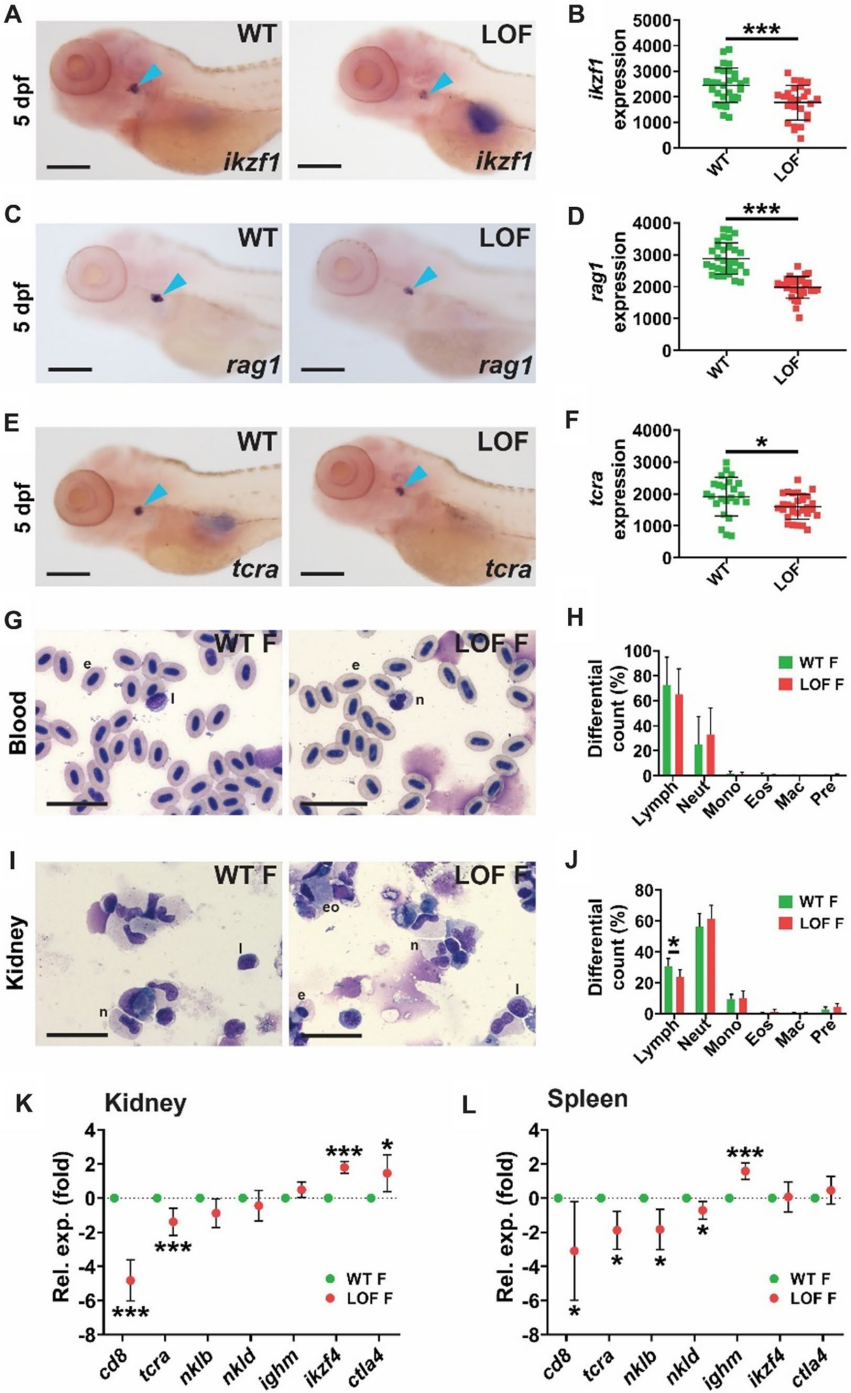
Effect of LOF Stat5.1 mutation on lymphoid cells. **A–F** Assessment of early lymphopoiesis in 5 dpf homozygous *stat5.1*^*wt/wt*^ wild-type (WT) and *stat5.1*^*mdu022/mdu022*^ loss-of-function (LOF) mutant embryos using WISH analysis, presenting representative individuals for *ikzf1* **(A)**, *rag1* **(C)** and *tcra* **(E)**, along with quantification of the relative area of expression for *ikzf1* **(B)**, *rag1* **(D)** and *tcra* **(F)**, showing results for individual embryos along with mean ± SD and statistical significance (*n* = 25–31). Scale bar = 200 μm. **G–J.** Analysis of blood and immune cells in adults, presenting images of representative Giemsa-stained blood **(G)** and kidney marrow **(I)** smears from 5 month post-fertilisation WT and LOF female fish and quantification of indicated cell populations in blood **(H)** and kidney marrow **(J)** showing mean and SD with statistical significance indicated (*n* = 6). Scale bar = 20 μm. **K–L** Expression analysis of immune genes in adults using qRT^2^-PCR on kidney marrow **(K)** and spleen **(L)** samples from 5 month post-fertilisation WT and LOF female fish. Data for the indicated genes were normalised to *actb* and represented as relative fold-change compared to WT fish, with mean ± SD shown and statistical significance indicated (*n* = 6)

Early T cell lymphopoiesis was assessed with the lymphocyte precursor marker, *ikzf1* [42], and the mature T cell markers, *rag1* [43] and *tcra* [44], using WISH on 5 dpf embryos. A reduced area of expression was observed for each of these markers in LOF compared to WT embryos (Fig. 3A–F).

Adult lymphopoiesis was examined by analysis of peripheral blood, kidney marrow, which plays a similar role as a mammalian bone marrow [36], and spleen. No significant differences in peripheral immune cell populations were observed in female (Fig. 3G–H) or male (Supp. Figure 1A–B) fish. However, a decreased proportion of lymphocytes was observed in female kidney marrow (Fig. 3I–J), although not in male kidney marrow (Supp. Figure 1C–D). Further analysis of female kidney marrow showed significantly decreased expression of T cell markers (*cd8*, *tcra*) but no change in markers for NK (*nklb*, *nkld*) and B cells (*ighm*) [45]. In contrast two T cell genes that mark both activated and Treg cells, *ikzf4* [46] and *ctla4* [47], were significantly increased (Fig. 3K). Similar results were observed in male kidney (Supp. Figure 1E). In the spleen, expression of *cd8*, *tcra* and *nklb* were significantly downregulated in both female and male LOF fish with *nkld* decreased just in female LOF compared to WT fish. Expression of *ighm* was increased in the spleens of both female and male LOF fish while expression of *ctla4* was significantly increased just in male LOF fish (Fig. 3L and Supp. Figure 1F).

## Discussion

STAT5B is an important transcription factor that plays a crucial role in cytokine signalling mediating somatic growth [3] and immune homeostasis [6]. STAT5B LOF mutations underpin an autosomal-recessive disorder characterised by severe growth failure and significant perturbation of lymphoid lineages, termed GHISID1 [7]. CRISPR/Cas9-based genome editing was used to successfully generate a zebrafish Stat5.1 mutant similar to known human STAT5B LOF mutations [16, 48]. Homozygous Stat5.1 mutant zebrafish showed defective growth and immune dysregulation, characterised by reduced size of both males and females, with the latter also displaying increased adiposity, along with reduced numbers of T lymphocytes throughout the life-course and alterations in other lymphocyte subsets in both sexes. No difference in fertilisation, survival or behavioural responses were noted (Supp. Figure 2 and data not shown). Moreover, heterozygous Stat5.1 mutant fish displayed no difference in lymphocyte numbers (Supp. Figure 3A-B) or growth (Supp. Figure 3C), confirming the autosomal-recessive nature of this mutation. Analysis of an alternative Stat5.1 C-terminal deletion mutant, in which the entire transactivation domain and tyrosine motif were absent (Supp. Figure 4A–D), confirmed the reduction in size in adults of both sexes and enhanced adiposity in females (Supp. Figure 4E–H) as well the reduction in embryonic T cells (Supp. Figure 4I–L). Morpholino-mediated knockdown further confirmed the reduced size and decreased T cells during embryogenesis (data not shown). These data collectively confirmed the creation of a zebrafish Stat5.1 LOF model that successfully mimicked human GHISID1.

The zebrafish Stat5.1 mutants showed a reduced growth trajectory during embryogenesis that continued into adulthood with both male and female mutant fish smaller than their wild-type counterparts despite increased *gh1* expression. This is consistent with patients harbouring LOF STAT5B mutations, with both males and females experiencing postnatal growth failure despite unaffected or elevated GH levels [16] and GH signalling shown to be directly impacted [14]. Stat5b knockout mice also showed reduced postnatal growth with elevated plasma GH, although this was limited to males where sexual dimorphic growth is strongly mediated by GH [4, 49]. Currently the number of patients with LOF STAT5B mutations is low, but as more are identified it will be of interest to uncover whether any phenotypic dimorphism exists between sexes. Either way these data confirm a conserved key role of STAT5B/Stat5.1 downstream of GHR, consistent with STAT5 binding sites being present on the duplicated zebrafish GHR proteins (Supp. Figure 5A). In humans the growth deficits that result from STAT5B LOF mutations are equivalent to those caused by GHR LOF mutations [7]. In contrast, a more severe growth defect was seen with a GH-deficient zebrafish than observed with the Stat5.1 LOF mutant [50]. This is similar to mice, where both *Gh*^*─/─*^ [51] and *Ghr *^*─ / ─*^ [52] mutants were smaller than *Stat5b*^*─/─*^ mutants [49], although disruption of both *Stat5a* and *Stat5b* genes imparted a more pronounced effect [49]. This suggests that Stat5a can partially compensate for Stat5b in mice, with Stat5.2 potentially able to do the same in zebrafish. *STAT5B* is expressed more highly than *STAT5A* [53], explaining why such compensation may not occur in humans.

In STAT5B LOF patients [13] and male Stat5b knockout mice [4, 49] the observed growth defects correlated with reduced IGF-I levels. In contrast, the zebrafish Stat5.1 LOF mutants showed a significant reduction in *igf2a*, but not *igf1, igf2b* or *igf3*. In mammals, IGF-2 is particularly involved in the regulation of foetal growth, being regulated by a complex imprinting mechanism [54]. In fish, however, this mechanism does not exist, conceivably allowing it to play a broader role. In fact it is the sole *igf* gene expressed in the embryonic zebrafish liver [39], while muscle *igf2a* has been shown to mediate the hypertrophy associated with GH transgenesis in zebrafish [55], tissues where *stat5.1* is strongly expressed (data not shown). In contrast, *igf1* appears to have a diminished role, with *igf1*-deficient zebrafish exhibiting only a mild size reduction [56]. RNAseq analysis of zebrafish Stat5.1 LOF mutant embryos (Table 1) identified down-regulation of two relevant pathways, ‘Insulin/IGF-MAPKK/MAPK’ and ‘Insulin/IGF-PKB’. Collectively, this suggests a core GH/GHR/STAT5/IGF pathway utilised in growth regulation across higher vertebrates, with species-specific diversification at the level of the individual proteins involved.

**Table 1:** Downregulated pathways in Stat5.1 LOF embryos

Gonadotrophin-releasing hormone receptor pathway (P06664)
CCKR signalling pathway (P06959)
PDGF signalling pathway (P00047)
Huntington's disease (P00029)
Angiogenesis (P00005)
Apoptosis (P00006)
Insulin/IGF pathway-protein kinase B signalling cascade (P00033)
Inflammation mediated by chemokine and cytokine signalling pathway (P00031)
Ras Pathway (P04393)
Integrin signalling (P00034)
Insulin/IGF pathway-mitogen activated protein kinase kinase/MAP kinase cascade (P00032)
Interleukin signalling (P00036)
Wnt signalling pathway (P00057)
Heterotrimeric G-protein Gi a and Gs a mediated pathway (P00026)

Eggs from Stat5.1 LOF mutants were smaller than those from WT fish. As expected, this correlated with having a Stat5.1 LOF mother regardless of the genotype of the father (Supp. Figure 6). This is consistent with previous studies that showed fish egg size was positively related to the size of the mother [57]. To date, no data are available on the birth weight of progeny from homozygous STAT5B LOF women. However, the mean birth weight of *Ghr *^*─/─*^ piglets was 40% lower when derived from *Ghr *^*─/─*^ rather than *Ghr* ^+*/─*^ mothers, attributed to reduced foetal growth capacity within the smaller *Ghr *^*─/─*^ mothers [58]. Therefore, impacts on foetal/egg size constitute an important alternate mechanism by which mutations in the GH/GHR/STAT5/IGF pathway can impact organism size.

Zebrafish Stat5.1 LOF mutants demonstrated greater adiposity. This was evident as increased lipid deposits in juveniles and elevated relative lipid content in adults, although the latter was restricted to females. The heightened adiposity correlated with a significant increase in expression of genes involved in lipid metabolism, including *srebf1*, and was consistent with a similarly enhanced adiposity observed in GH-deficient zebrafish [50]. Several patients harbouring STAT5B LOF mutations also displayed central obesity [15, 48], while those with acquired GH deficiency possess increased visceral and abdominal fat mass in particular [59, 60]. Stat5b knockout mice [4], as well as mice expressing a truncated GHR unable to activate STAT5 [61], exhibited a similar increase in adiposity, with Stat5b-deficient mice also shown to have elevated *Srebf1* expression [62]. In combination, these observations indicate a conserved role for STAT5B/Stat5.1 downstream of GHR in regulating key genes involved in lipid metabolism, with enhanced adiposity resulting from disruption of this pathway.

Zebrafish Stat5.1 LOF mutants showed decreased numbers of lymphocyte precursors and T lymphocytes during embryogenesis and reduced T lymphocytes in adulthood, with splenic NK cells also reduced. Patients carrying STAT5B LOF mutations, including those harbouring a R152X mutation, exhibited lymphoid defects [16, 63], with reduced numbers of T cells (CD4^+^, CD8^+^), NK cells and Treg cells [48, 63–65], with signalling by IL-2 cytokines shown to be affected [66]. Stat5b knockout mice also possessed a subtle reduction in the number and proliferation of T lymphocytes [67] as well as reduced NK cell number and function [68, 69]. In each case the impact on lymphoid cells was similar but less severe than observed with mutation of the common IL-2R gamma chain, which mediates signalling for the IL-2 cytokine family in humans [70], mice [71] and zebrafish [72]. This might be due to redundancy with STAT5A/Stat5.2, since Stat5a^─/─^/Stat5b^─/─^ double mutant mice exhibited a more severe immune phenotype [49], but also likely the result of alternate STAT proteins and indeed other pathways activated in parallel [73]. We have previously demonstrated Stat5.1 acting downstream of zebrafish II-2rγc [74] and Jak3 [74, 75], with conserved STAT5 docking sites on the II-2rβ receptor chain that utilises II-2rγc (Supp. Figure 5B). Moreover, RNAseq analysis confirmed down-regulation of the ‘Interleukin signalling’ as well as the ‘Inflammation mediated by chemokine and cytokine signalling’ pathways (Table 1), while *stat5.1* was expressed in the major zebrafish haematopoietic organ, the kidney. Together this provides further evidence of a conserved IL-2Rγc/JAK3/STAT5 pathway in lymphopoiesis. Zebrafish Stat5.1 LOF mutants additionally showed increased expression of the gene encoding IgM as well as markers of activated T cells [46, 47]. This is in line with some STAT5B LOF human patients that show high levels of IgG and activated T cells along with autoimmune disease [63, 64]. Further analysis of this aspects of the clinical phenotype appears worthwhile.

An alternate zebrafish Stat5.1 LOF mutant has been described that also showed a similar reduction in growth to that reported here, although this was associated with reduced GH expression and loss of female-biased size dimorphism but not increased female adiposity [76]. It remains unclear why such different phenotypes were observed. Further analysis of this mutant revealed loss of sexually dimorphic gene expression, with multiple pathways related to metabolism affected [77]. The immune system was not investigated in this alternate mutant. However, a strong interplay between metabolism and immunity has been identified [78] providing a potential additional mechanism to explain the impacts of STAT5B/Stat5.1 mutations. A range of other clinically important mutations impacting cytokine receptor signalling via the JAK-STAT pathway have been identified [79]. Our success of modelling STAT5B-mediated GHISID1 in this study, as well as IL-2Rγc-mediated SCID [72] and gain-of-function (GOF) mutations of JAK2 [80], JAK3 [75] and STAT5B [81] associated with haematological malignancy, along with the work of others investigating the impact of LOF STAT3 mutations [82], highlight the strong potential for using zebrafish to explore the role of JAK-STAT perturbation in disease. We hope to extend this research to autosomal-dominant STAT5B mutations associated with GHISID2 [17], as well as GOF STAT3 mutations implicated in immune dysregulation [83], and to utilise these platforms for therapeutic testing.

### Supplementary Information

Below is the link to the electronic supplementary material.

Supplementary file1 (PDF 1744 KB)

## Data Availability

All data generated or analysed during this study are included in this article (and its Supplementary Material files).

## Abbreviations

dpf: Days post-fertilisation
GH: Growth hormone
GHISID: Growth hormone insensitivity syndrome with immune dysregulation
GOF: Gain-of-function
gRNA: Guide ribonucleic acid
HRM: High resolution melt
IGF: Insulin-like growth factor
IL: Interleukin
IL-2Rγc: IL-2 receptor gamma common
LOF: Loss-of-function
mpf: Months post-fertilisation
PCR: Polymerase chain reaction
PRL: Prolactin
qRT^2^-PCR: Quantitative real-time reverse-transcription-PCR
R: Receptor
SD: Standard deviation
STAT: Signal transducer and activator of transcription
WISH: Whole-mount in situ hybridisation
WT: Wild-type
