# Supplementary material for: A zebrafish model of growth hormone insensitivity syndrome with immune dysregulation 1 (GHISID1)
Source: Cell Mol Life Sci. 2023 Mar 30;80(4):109. doi: 10.1007/s00018-023-04759-y (PMC10063521; doi:10.1007/s00018-023-04759-y)

Supp. Fig. 1 Effect of LOF Stat5.1 mutation on lymphoid cells in adult male fish

A-D. Analysis of blood and immune cells in adults, presenting images of representative Giemsa-stained blood **(A)** and kidney marrow **(C)** smears from 5 month post-fertilization *stat5.1*$^{wt/wt}$ wild-type (WT) and *stat5.1*$^{mdu022/mdu022}$ loss-of-function (LOF) male (M) fish and quantification of indicated cell populations in blood **(B)** and kidney marrow **(D)**, showing mean and SD with statistical significance indicated (n=6)**.** Scale bar = 20 μm.

E-F. Expression analysis of immune genes in adults using qRT^2-PCR on kidney marrow **(E)** and spleen **(F)** samples from 5 month post-fertilization WT and LOF male fish. Data for the indicated genes was normalized relative to *actb* and represented as relative fold-change compared to WT fish, with mean ± SD shown and statistical significance indicated (n=6).

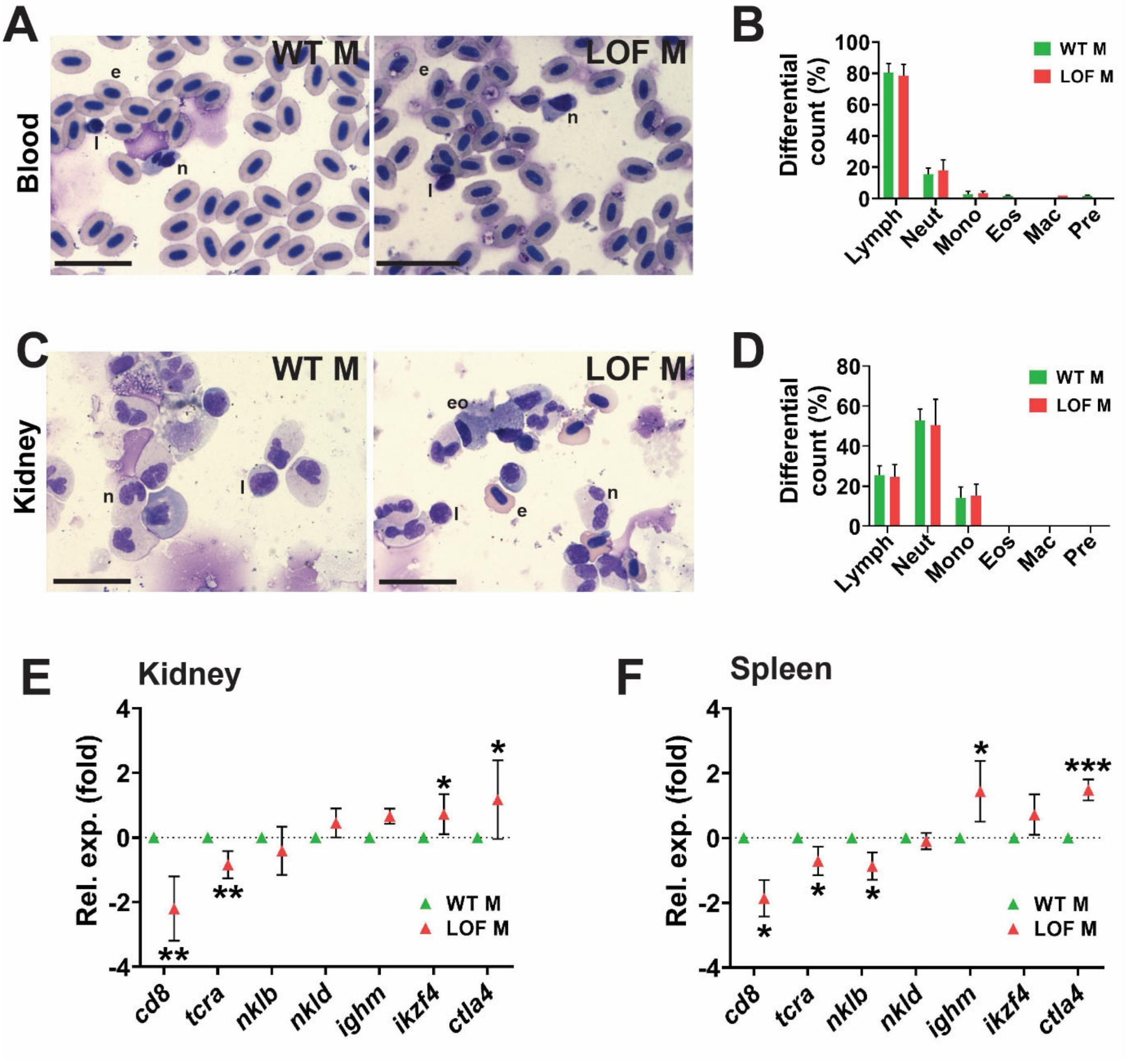

Supp. Fig. 2 Analysis of other potential impacts of Stat5.1 mutation

Analysis of progeny of *stat5.1*$^{wt/wt}$ wild-type (WT) and *stat5.1*$^{mdu022/mdu022}$ loss-of-function (LOF) mutant fish with respect to fertilization rate (A), embryo survival to 7 dpf (B) and adult survival at 7 mpf (C). Panels A and C show values for individual embryos as well as mean ± SD and panel B is a Kaplan-Meier plot, with statistical significance indicated in each case.

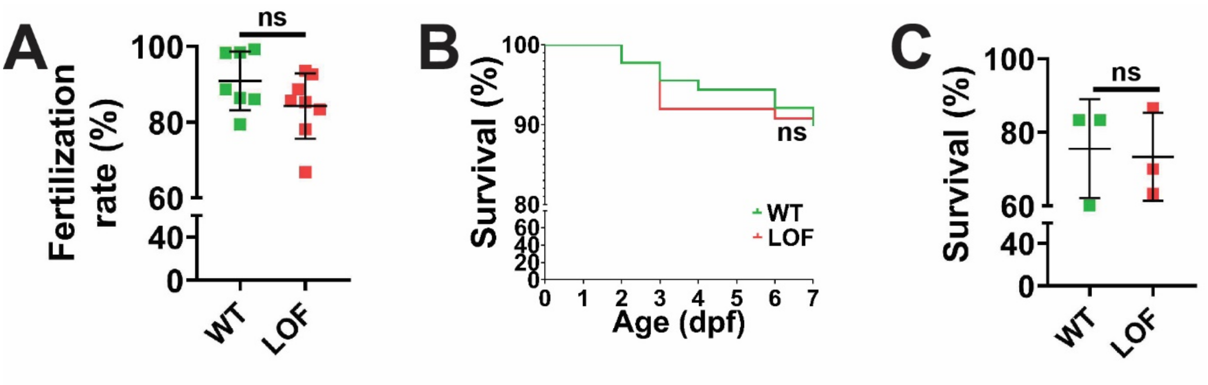

Supp. Fig. 3 Analysis of heterozygous carriers of LOF Stat5.1 mutation with respect to lymphoid cells and growth

A-B. Assessment of early lymphopoiesis in 5 dpf homozygous *stat5.1*$^{wt/wt}$ wild-type (WT) and *stat5.1*$^{wt/mdu022}$ heterozygous (HET) embryos using WISH analysis with *rag1*, presenting representative individuals **(A)** along with quantification of the relative expression for *rag1* **(B)**, showing results for individual embryos along with mean ± SD and statistical significance indicated (n=24-28). Scale bar = 200 µm.

C. Assessment of size for WT and HET fish at 2 month post-fertilization, showing values for individual fish as well as mean ± SD with statistical significance indicated (n=7).

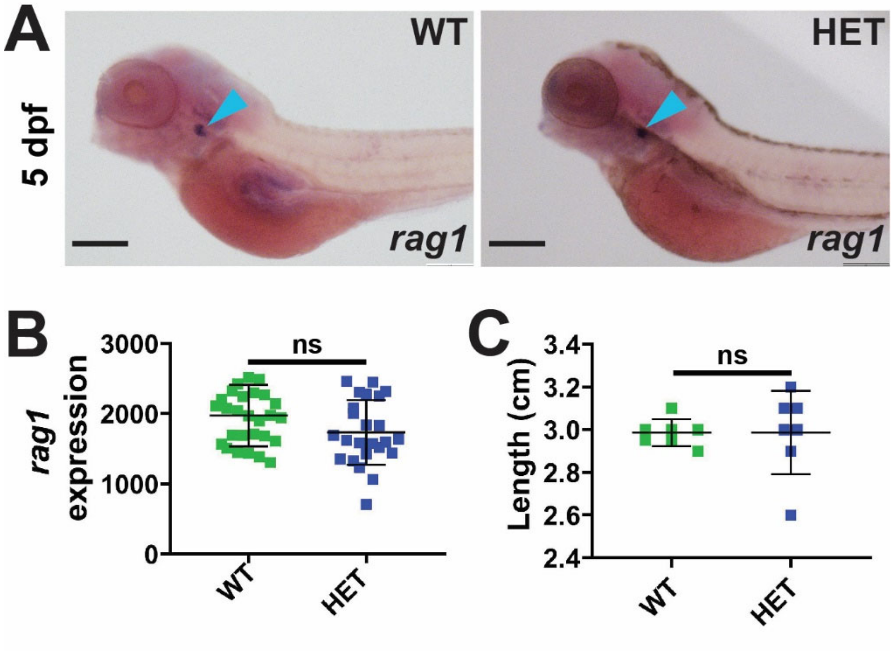

Supp. Fig. 4 Analysis of alternative Stat5.1 mutant

A-D. Schematic diagram of the STAT5B protein domains **(A)**, with alignment of human STAT5B with zebrafish Stat5.1 protein sequences around the mutation site **(B)**, nucleotide sequence of zebrafish *stat5.1* genomic DNA targeted using CRISPR/Cas9 **(C)**, along with sequence of homozygous wild-type (WT, *wt/wt*) and C-terminal deletion (ΔC, *mdu031/mdu031*) mutant *stat5.1*, 12 bp deletion and 4 bp insertion (pink text) and nine *de novo* amino acid residues and stop codon (orange text) in the ΔC mutant **(D)**, with other details as described in Figure 1 legend.

E-H. Assessment of adult size and body composition in homozygous $stat5.1^{wt/wt}$ wild-type (WT) and $stat5.1^{mdu031/mdu031}$ C-terminal deletion (ΔC) mutants at 5 months post-fertilization, presenting representative images **(E)** along with quantification of standard length **(F)** and wet weight **(G)** for female (F) and male (M) fish, along with lipid content for females **(H),** showing values for individual fish as well as mean ± standard deviation (SD) with statistical significance indicated (n=6).

I-L. Assessment of early lymphopoiesis in 5 dpf homozygous $stat5.1^{wt/wt}$ wild-type (WT) and $stat5.1^{mdu031/mdu031}$ C-terminal deletion (ΔC) mutant embryos using WISH analysis, presenting representative individuals for *rag1* **(I)** and *tcra* **(K)**, along with quantification of the relative area of expression for *rag1* **(J)** and *tcra* **(L)**, showing results for individual embryos along with mean ± SD and statistical significance indicated (n=20-25). Scale bar = 200 μm.

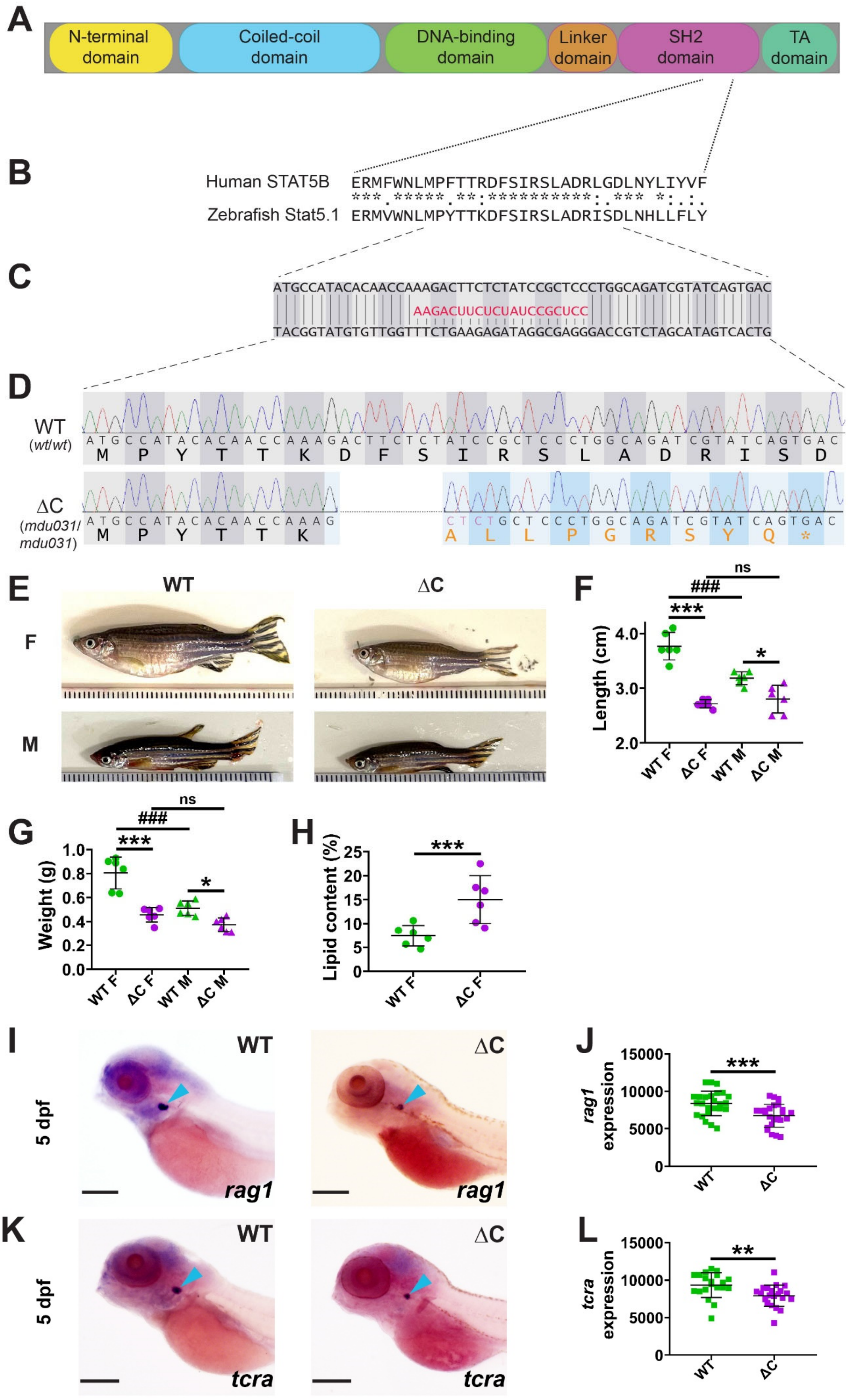
A
N-terminal domain
Coiled-coil domain
DNA-binding domain
Linker domain
SH2 domain
TA domain
B
Human STAT5B ERMFWNLMPFTTRDFSIRSLADRLGDLNYLIYVF
Zebrafish Stat5.1 ERMVWNLMPYTTKDFSIRSLADRISDLNHLLFLY
C
ATGCCATACACAACCAAAGACTTCTCTATCCGCTCCCTGGCAGATCGTATCAGTGAC
AAGACUUCUCUAUCCGCUCC
TACGGTATGTGTTGGTTTCTGAAGAGATAGGCGAGGGACCGTCTAGCATAGTCACTG
D
WT (wt/wt)
M P Y T T K D F S I R S L A D R I S D
ΔC (mdu031/ mdu031)
M P Y T T K
A L L P G R S Y Q *
E
WT
ΔC
F
M
F
Length (cm)
WT F
ΔC F
WT M
ΔC M
G
Weight (g)
H
Lipid content (%)
I
5 dpf
rag1
J
rag1 expression
WT
ΔC
K
tcra
L
tcra expression

Supp. Fig. 5 Conservation of STAT5 docking sites

Alignment of sequences around key STAT5 docking sites for human (Hs) and zebrafish (Dr) growth hormone receptor (GHR) (A) and interleukin-2 receptor beta (IL2RB) (B), with tyrosine residue highlighted. * = identical; : = very similar; . = similar.

A

```
Dr Ghrb (408) SNNLYSQVSDI
              . ::*:*****
Hs GHR  (487) NIDFYAQVSDI
              *:********:
Dr Ghra (434) NMDFYAQVSDF
```

B

```
Hs IL2RB (536) NTDAYLSLQEL
               . ..**:*:*:
Dr Il2rb (489) SGSGYLTLKEM
```

Supp. Fig. 6 Dependence of yolk size on parental Stat5.1 genotype

Quantitation of yolk diameter of 1 cell embryos derived from the indicated crosses of *stat5.1*$^{wt/wt}$ wild-type (WT) and *stat5.1*$^{mdu022/mdu022}$ loss-of-function (LOF) mutant female (F) and male (M) fish, showing values for individual embryos as well as mean ± SD with statistical significance indicated (n=24).

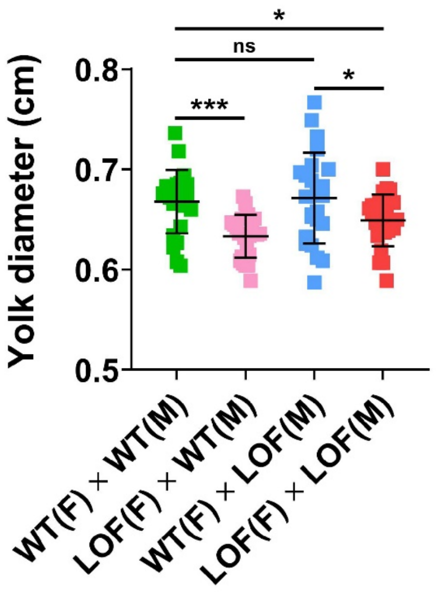

Supplement: Supplementary file 1 — Supplementary file1 (PDF 1744 KB) [file 18_2023_4759_MOESM1_ESM.pdf]
